# Spatial distribution and environmental correlations of *Culex pipiens pallens* (Diptera: Culicidae) in Haidian district, Beijing

**DOI:** 10.1093/jme/tjae063

**Published:** 2024-05-15

**Authors:** Meide Liu, Yong Zhang, Qiuhong Li, Xiaojie Zhou, Ting Yan, Jing Li, Hongjiang Zhang, Lei Wang, Guangwen Wang, Ruoxi Li, Ying Tong, Xiaopeng Zeng

**Affiliations:** Institute for Disinfection and Vector Control, Beijing Municipal Center for Disease Prevention and Control, 16 Hepingli Zhong Street, Dongcheng District, Beijing 100013, China; Institute for Disinfection and Vector Control, Beijing Municipal Center for Disease Prevention and Control, 16 Hepingli Zhong Street, Dongcheng District, Beijing 100013, China; Institute for Disinfection and Vector Control, Beijing Municipal Center for Disease Prevention and Control, 16 Hepingli Zhong Street, Dongcheng District, Beijing 100013, China; Institute for Disinfection and Vector Control, Beijing Municipal Center for Disease Prevention and Control, 16 Hepingli Zhong Street, Dongcheng District, Beijing 100013, China; Institute for Disinfection and Vector Control, Beijing Municipal Center for Disease Prevention and Control, 16 Hepingli Zhong Street, Dongcheng District, Beijing 100013, China; Institute for Disinfection and Vector Control, Beijing Municipal Center for Disease Prevention and Control, 16 Hepingli Zhong Street, Dongcheng District, Beijing 100013, China; Institute for Disinfection and Vector Control, Beijing Municipal Center for Disease Prevention and Control, 16 Hepingli Zhong Street, Dongcheng District, Beijing 100013, China; Department of Disinfection and Sanitation, Haidian District Center for Disease Control and Prevention, Beijing 100037, China; Department of Disinfection and Vector Control, Fangshan District Center for Disease Control and Prevention, Beijing 102446, China; Department of Disinfection and Vector Control, Fengtai District Center for Disease Control and Prevention, Beijing 100068, China; Institute for Disinfection and Vector Control, Beijing Municipal Center for Disease Prevention and Control, 16 Hepingli Zhong Street, Dongcheng District, Beijing 100013, China; Institute for Disinfection and Vector Control, Beijing Municipal Center for Disease Prevention and Control, 16 Hepingli Zhong Street, Dongcheng District, Beijing 100013, China

**Keywords:** *Culex pipiens pallens*, spatial distribution, environmental factor, urban area

## Abstract

*Culex pipiens pallens* Coquillett, 1898 (Diptera: Culicidae) was the dominant health threat to mosquito species in Beijing, and it is important to unravel the spatial distribution and environmental correlations of *Cx. pipiens pallens* in Beijing. 3S technology methods and spatial statistics were used to clarify the distribution pattern. Subsequently, linear and spatial regression were performed to detect the environmental factors linked with the density of *Cx. pipiens pallens*. The same “middle peak” spatial distribution pattern was observed for *Cx. pipiens pallens* density at the community, subdistrict, and loop area levels in our study area. In addition, there were various correlated environmental factors at the community and subdistrict scales. At the community scale, the summary values of the Modified Normalized Difference Water Index (MNDWI) in 2 km buffer zone (MNDWI_2K) were negatively correlated, and the summary values of Normalized Difference Built-up Index (NDBI) in 800 m buffer zone (NDBI_800) was positively correlated to the *Cx. pipiens pallens* density. However, the summary values of Normalized Difference Vegetation Index and Nighttime Light Index significantly affected *Cx. pipiens pallens* density at the subdistrict scale. Our findings provide insight into the spatial distribution pattern of *Cx. pipiens pallens* density and its associated environmental risk factors at different spatial scales in the Haidian district of Beijing for the first time. The results could be used to predict the *Cx. pipiens pallens* density as well as the risk of lymphatic filariasis (LF) infection, which would help implement prevention and control measures to prevent future risks of biting and LF transmission in Beijing.

## Introduction

In Beijing, *Cx. pipiens pallens* accounts for almost 90% of the local mosquito community, and biting is its major infestation for humans (Meide et al. 2019). With the effects of global climate change, the risk of spread of the vector and its transmitted disease will also rise in coming years (Lourens and Ferrell 2019, Folly et al. 2021) Urban areas provide beneficial waterbodies (Castro et al. 2010), channel (Mogi and Sota 1996) and container (Townroe and Callaghan 2014) for *Cx. pipiens pallens* breeding, which results in high adaptation of *Cx. pipiens pallens* to urban environments and the potential risk of West Nile virus and lymphatic filariasis (LF) in these settings (Nchoutpouen et al. 2019). The predominance of *Cx. pipiens pallens* in urban mosquito communities (Meide et al. 2019), as well as a higher density of *Cx. pipiens pallens* (Yuan et al. 2012) and suspected clinical cases of LF (Terranella et al. 2006) have been observed in urban areas. Considering the public and economic burden of chronic LF disease in urban areas, it is necessary to conduct *Cx. pipiens pallens* surveillance and control in urban settings, given the rapid growth of urban areas (Simonsen and Mwakitalu 2013). Moreover, the spatial distribution of *Cx. pipiens pallens*, and other mosquitoes was also found to be driven by environmental features (Cardo et al. 2016). Therefore, identifying the link between environmental features and *Cx. pipiens pallens* density is important for understanding the spatial or temporal distribution pattern of this mosquito species in urban areas.

From the viewpoint of vector ecology, urban waterbodies (Castro et al. 2010) and vegetation (Gardner et al. 2017) are key features impacting the ecology of *Cx. pipiens pallens* in cities. For instance, the remotely sensed Normalized Difference Vegetation Index (NDVI) was used to identify parameters associated with the aquatic habitats of field-sampled *Cx. pipiens pallens* (Jacob et al. 2009). In addition, the effects of urbanization on the *Cx. pipiens pallens* population has been demonstrated in many studies (Zarechnaia et al. 1989). As the key indices of urbanization, the remotely sensed Normalized Difference Built-up Index (NDBI) and Nighttime Light Index (NLI) must be closely associated with the spatial distribution of *Cx. pipiens pallens* in an urban setting. Many studies have focused on the relationship between the built-up landscape (Field et al. 2019) and *Culex* vector populations in urban regions (Deichmeister and Telang 2011). At the same time, the NLI, which is a typical urbanization index, was seldom used to link the density or distribution of *Cx. pipiens pallens* and urban settings.

Implementing appropriate mosquito management strategies is dependent on understanding the abundance, distribution, species composition, and mosquito interactions during disease transmission in endemic areas (Mulyaningsih et al. 2019). In urban settings, the *Cx. pipiens pallens* distribution and density could be modeled and predicted using many environmental features, including vegetation and waterbody characteristics (Gardner et al. 2013). GIS and remote sensing have been confirmed to perform well in the monitoring and prediction of *Cx. pipiens pallens* and its transmission of LF (Mwase et al. 2014) and for quantification and model analysis on larger geographical scales (Sowilem et al. 2006). Thus, 3S methods were adapted in this study to quantify the environmental factors, conduct spatial analyses at different spatial scales, and model the relationship between the *Cx. pipiens pallens* density and environmental factors.

To date, no studies have examined the urban spread pattern of *Cx. pipiens pallens* and its background environmental characteristics in the Beijing urban region or modeled or predicted its distribution based on environmental datasets. Thus, this study aimed to examine the spatial distribution and environmental correlations of *Cx. pipiens pallens* in the urban setting of Beijing. Based on the relationship between the mosquito and the environmental background, *Cx. pipiens pallens* in the Beijing urban area must display particular correlation and modeling relationships. In the present study, we used 3S tools to analyze the correlation of environmental features with the spatial location of the *Cx. pipiens pallens* population and to model the *Cx. pipiens pallens* density with environmental features at the community and subdistrict scales. The Haidian district, our study area, lies in the northwestern part of Beijing, and its administrative division ranges from the center to the suburbs of Beijing. Thus, this study area represents the urban environment as well as the ecological characteristics of the mosquito population at the Beijing city level. Given the predominance of *Cx. pipiens pallens* in the mosquito community and its potential LF transmission risk, this study could provide field evidence to support the control of *Cx. pipiens pallens* or LF epidemiology in Beijing.

## Materials and Methods

### Study Area

**Fig. 1.**
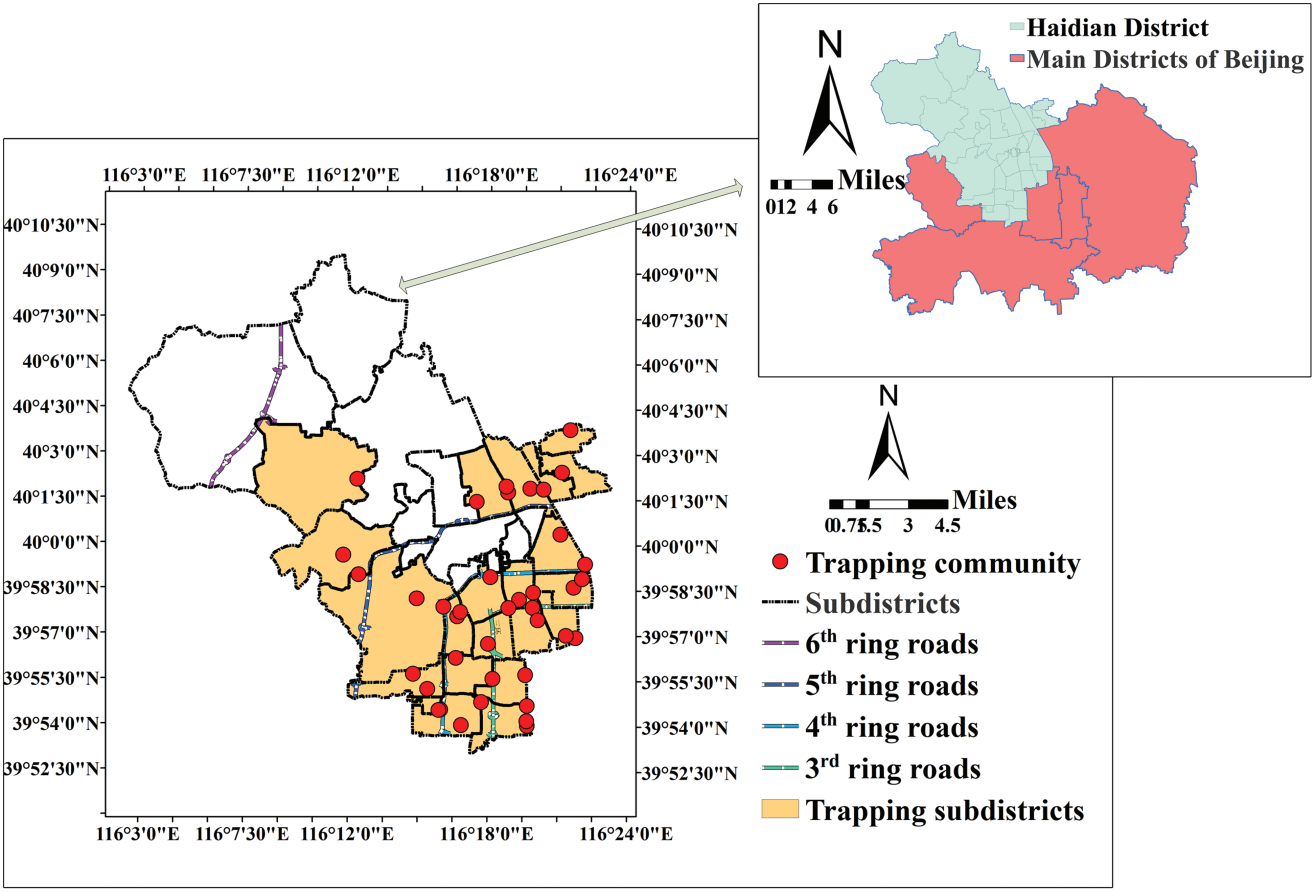
The map of mosquito trapping community and subdistrict.

The Haidian district lies in the northwestern part of Beijing City and encompasses an area of nearly 431 km^2^, which covers 2.6% of the Beijing area. The southern part of Haidian is a typical mountain area with an average altitude above 100 m that covers 15% of the district, while the southeastern part of the district is a typical plain area with an average altitude below 50 m that covers 85% of the district. There are 10 rivers totaling 119.8 km in length in the district, along with Kunming Lake and Yuyuantan and Shanzhuang water reserves, which provide rich water resources in this district. The 3rd, 4th, 5th, and 6th ring roads in Beijing run across the Haidian district and subdivide it into 4 loop areas (LAs) (named LA1, LA2, LA3, and LA4) extending from the city center to the suburbs of Haidian district (Fig. 1). The economic aggregate of Haidian district ranked first among the districts of Beijing, and Haidian district was designated as an urban functional development area (UFDA) according to the main functional area planning of Beijing in 2012, but its administrative regions cover the UFDA and the capital functional core area (CFCA) of Beijing.

### Mosquito Density Survey With Light Trapping

In this study, the Haidian district was selected as the study area and the density of *Cx. pipiens pallens* was surveyed at the community level. There are 29 subdistricts in Haidian district, and 20 of them were selected for trapping. In these trapping subdistricts, 39 communities were selected as mosquito survey sites. In Beijing, *Cx. pipiens pallens* could be seen in May–October, and the density peak of mosquitoes was observed in July and August. Thus, the survey was carried out in July and August, and the mosquitoes were trapped continuously over a 7-day period in the middle of each month. In our study area, at least 1 community in each subdistrict was selected, and 4 light trapping sites were located in each community. In total, 156 light trap sites, 39 communities, and 20 subdistricts were sampled in this study. Mosquitoes were trapped with light traps with carbon dioxide (MD-1 CO_2_ mosquito light trap 6 V/4.5AH, Beijing Logon Sic. & Tech. Co., Ltd, Beijing, China). At each light trapping site, 1 light trap was hung at 1.5 m from the ground, and the light was turned on at 7:00 PM and off at 7:00 AM daily. On bad weather days, trapping was postponed by 1 day. Every day, the mosquito samples were collected and transported to the laboratory for later identification, and the trapped female mosquitoes were identified by morphology (Lu 1997) and counted. In each trapping community, a GPS device was used to mark the position of the community and location data were applied to order satellite datasets over the study area.

### Calculation of Mosquito Density

The mosquito density (*N*) was calculated from the light trap density as follows:


$$\begin{array}{l} N=\frac{MN}{LN} \end{array}$$

where MN is the number of trapped mosquitoes, LN is the number of light traps, and the unit of mosquito density is the number of mosquitoes per trap (NMPT).

### Remote Sensing Analysis and Raster Map Production of the MNDWI, NDVI, and NDBI

Based on the Landsat ETM satellite datasets over Beijing city, the Modified Normalized Difference Water Index (MNDWI), NDVI, and NDBI raster maps were calculated using the following procedures:

The MNDWI was calculated according to (Xu 2005):


$$\begin{array}{l} MNDWI=\frac{\left(Green-MIR\right)}{\left(Green+MIR\right)} \end{array}$$

where Green and MIR stand for the spectral reflectance measurements acquired in the green (Green) and middle-infrared (MIR) regions, respectively.

The NDVI was calculated from these individual measurements as (TUCKER et al. 1986):


$$\begin{array}{l} NDVI=\frac{\left(NIR-RED\right)}{\left(NIR+RED\right)} \end{array}$$

where RED and NIR stand for the spectral reflectance measurements acquired in the red (RED) and near-infrared (NIR) regions, respectively.

The NDBI was calculated from these individual measurements as (Cha et al. 2003):


$$\begin{array}{l} NDBI=\frac{\left(MIR-NIR\right)}{\left(MIR+NIR\right)} \end{array}$$

where MIR and NIR stand for the spectral reflectance measurements acquired in the middle-infrared (MIR) and near-infrared (NIR) regions.

### Light Index Map

The light index map was downloaded from the NOAA website (http://ngdc.noaa.gov/eog/viirs/download_viirs_ntl.html) on the scale of a year, and the light index map of Beijing city was produced by intersecting the Beijing administration vector file with the light index map downloaded from NOAA.

### Construction of Mosquito and Environmental Spatial Datasets at the Community and Subdistrict Levels

At the subdistrict level, the average values of the MNDWI, NDVI, NDBI, and light index in each subdistrict area were extracted with spatial analysis in the context of QGIS (3.28). At the community level, the geographic center of each community was first identified as a community site. Using the buffer tool in QGIS (3.28), circular buffer zones of different sizes (100, 200, 400, 800, 1,000, 2,000, 3,000, 4,000, and 5,000 m) were drawn around each community site based on the TM image datasets. The summary values of the MNDWI, NDVI, NDBI, and light index in each buffer zone of each community site were extracted with spatial analysis in the context of QGIS (3.28). Subsequently, spatial datasets of subdistricts, including the *Cx. pipiens pallens* density and MNDWI, NDVI, NDBI, and light index values, were compiled in QGIS (3.28), which were the datasets used in the correlation analysis of mosquito density and environmental factors.

### Statistical Correlation and Regression Analysis of Mosquito Density With Environmental Factors at the Community Level

Mosquito density at the community level was determined using the following steps. First, the mosquito trapping data from the same community were synthesized as the trapping dataset for the community. Then, correlation analysis of *Cx. pipiens pallens* density with environmental factors was performed in SPSS (15.0) to detect the possible factors correlated to mosquito density. Finally, environmental factors that were significantly correlated with mosquito density were introduced into the regression analysis of *Cx. pipiens pallens* density with environmental factors.

### Spatial Autocorrelation Test and Spatial Regression of Mosquito Density at the Subdistrict Level

First, the trapping data at the subdistrict level were synthesized for the trapping sites located in the same subdistrict. After that, global and local Moran’s *I* tests were performed to study the spatial aggregation characteristics with GeoDa (1.14.0). The positive Moran’s *I* indicate spatial clustering, the negative Moran’s *I* suggest diffusion pattern, and the zero Moran’s *I* point to spatial random scattering. Finally, spatial regression was also used to detect the link between mosquito density and environmental features at the subdistrict level.

Before spatial regression, the order of each environmental factor included in the spatial regression was decided based on absolute values of the standardized coefficient (Beta) of the individual factor to the *Cx. pipiens pallens* density. Then, the factors were introduced in the regression model one by one by the ranks of the absolute values of the standardized coefficient (Beta) from large to small, which resulted in many regression model frameworks. For each model framework, 3 regression methods (nonspatial, spatial lag, and spatial error) were applied to obtain the AIC and R-squared values that could be used to decide on the best model.

### Comparison of Mosquito Density Among LAs

*Cx. pipiens pallens* density was compared among the LAs, and each community site was grouped according to their spatial location in the individual LA. Afterward, the mean *Cx. pipiens pallens* density value of the 4 LA groups was compared using one-way ANOVA followed by the multiple comparisons LSD test.

### Statistical Analysis

The statistical analysis was conducted with IBM SPSS Statistics for Windows, version 19.0 (IBM Corp., Armonk, NY, USA), and the statistical significance was set at *P* < 0.05.

## Results

### Spatial Distribution and Environmental Correlations at the Community Level

**Fig. 2.**
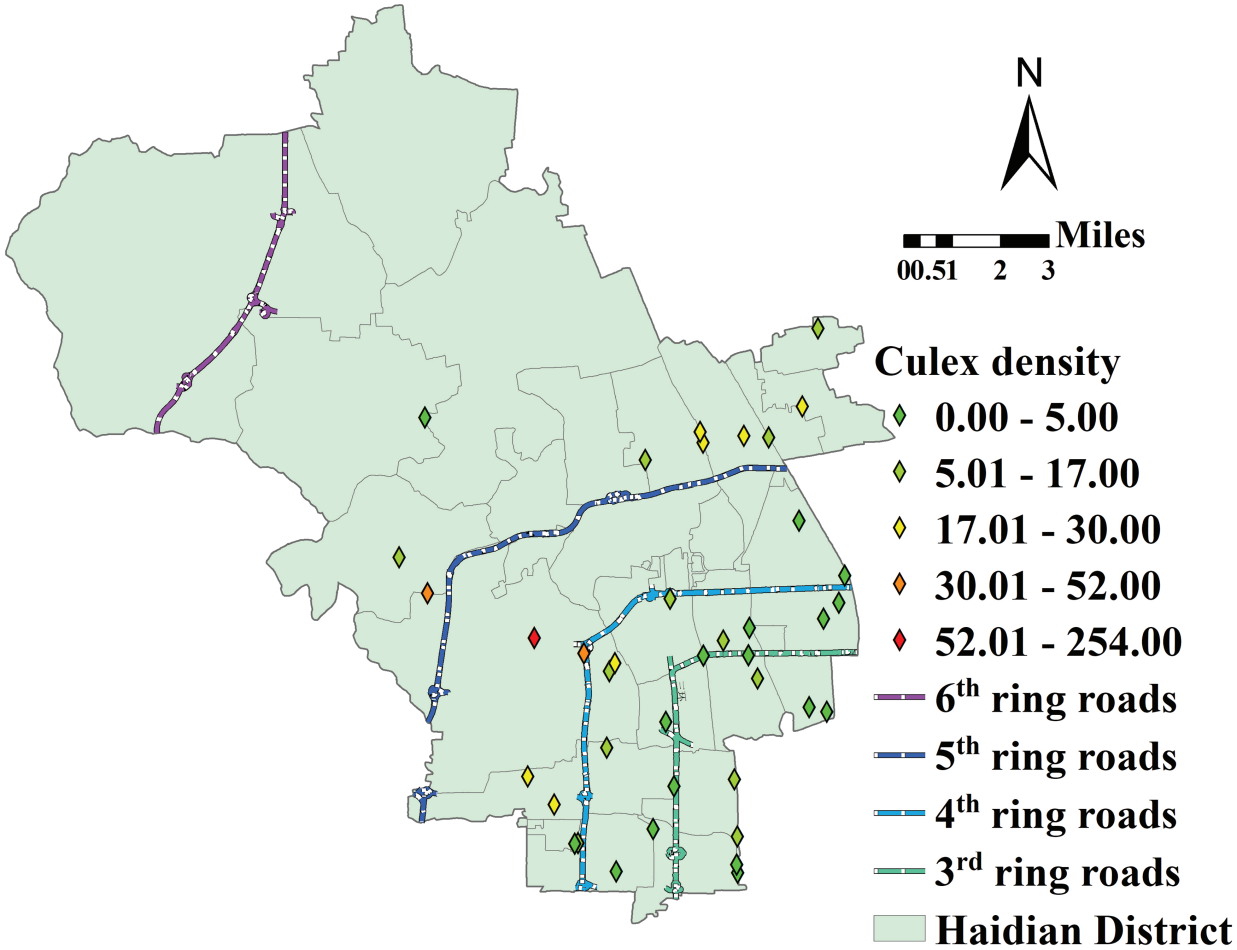
The spatial distribution pattern of *Cx. pipiens pallens* density on the level of community.

The communities with the highest *Cx. pipiens pallens* densities were primarily located in the middle and outer LA areas and those with lowest *Cx. pipiens pallens* densities were preferentially located in the center LA area (Fig. 2). Correlation and regression analysis showed that MNDWI_2K and NDBI_800 were significantly and linearly correlated with mosquito density. As the regression analysis showed, MNDWI_2K was negatively correlated with *Cx. pipiens pallens* density (−2.692 × 10^−6^, *P* = 0.0002), while NDBI_800 was positively correlated with *Cx. pipiens pallens* density (1.482 × 10^−5^, *P* = 0.0148) at the community scale. Thus, the linear model for *Cx. pipiens pallens* density was as follows: *D* = 2.573784 + 1.482 × 10^−5^ × NDBI_800-2.692 × 10^−6^ × MNDWI_2K (*R* = 0.630237, *F* = 11.860574, *P* = 0.0001, and *D* was the density of *Cx. pipiens pallens*).

### Spatial Distribution and Environmental Correlations at the Subdistrict Level Mosquito Density Spatial Distribution Among Subdistricts

**Fig. 3.**
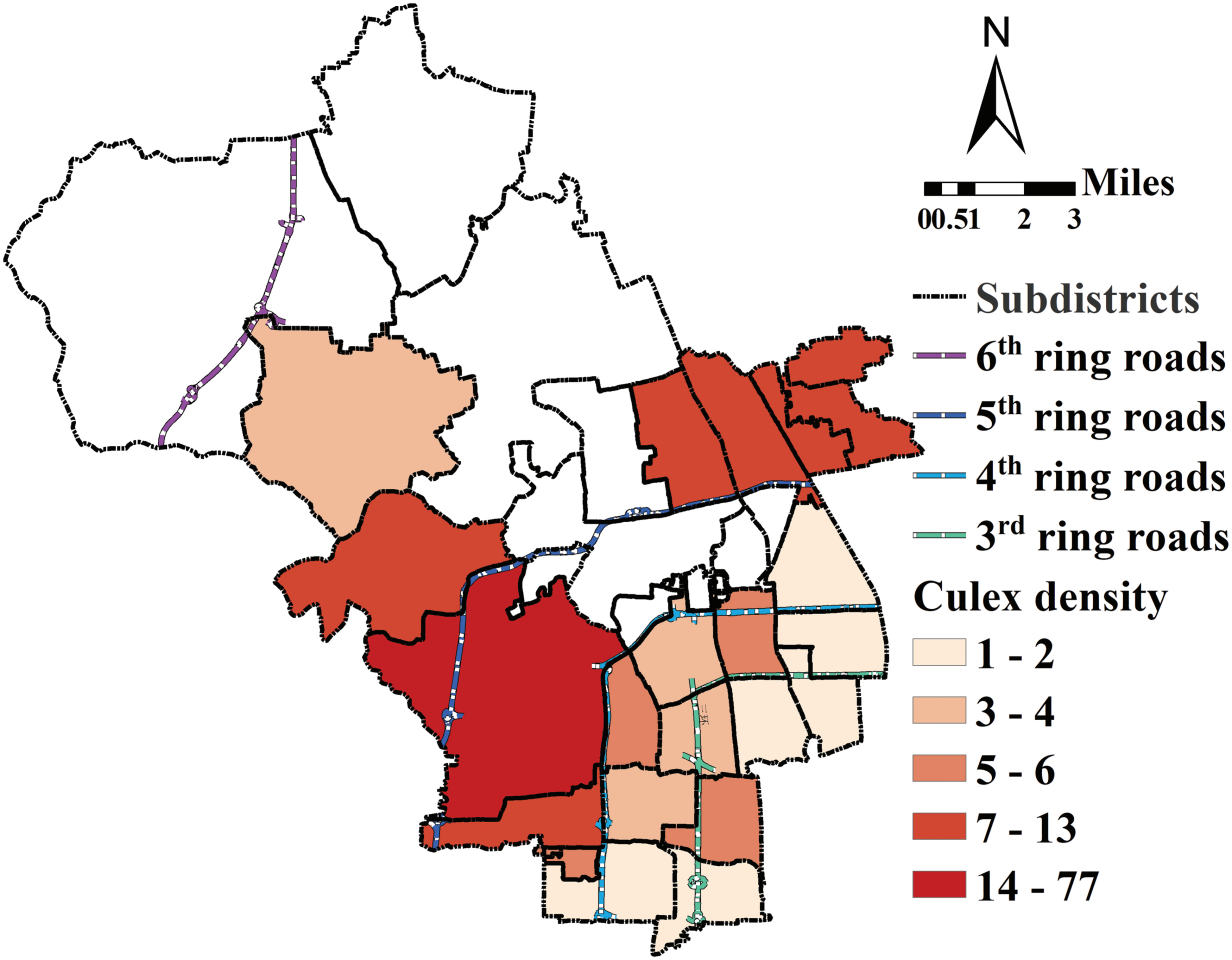
The density distribution of *Cx. pipiens pallens* on the level of the subdistrict.

As shown in Fig. 3, the density distribution of *Cx. pipiens pallens* showed a “middle peak” pattern. The density of *Cx. pipiens pallens* in the subdistrict near the city center was the lowest among the subdistricts, the density of *Cx. pipiens pallens* in the subdistrict farthest from the center city was intermediate, and the density of *Cx. pipiens pallens* in the subdistricts located in the middle was highest.

### Global Moran’s *I* Test of Spatial Autocorrelation of Mosquito Density at the Subdistrict Level

**Fig. 4.**
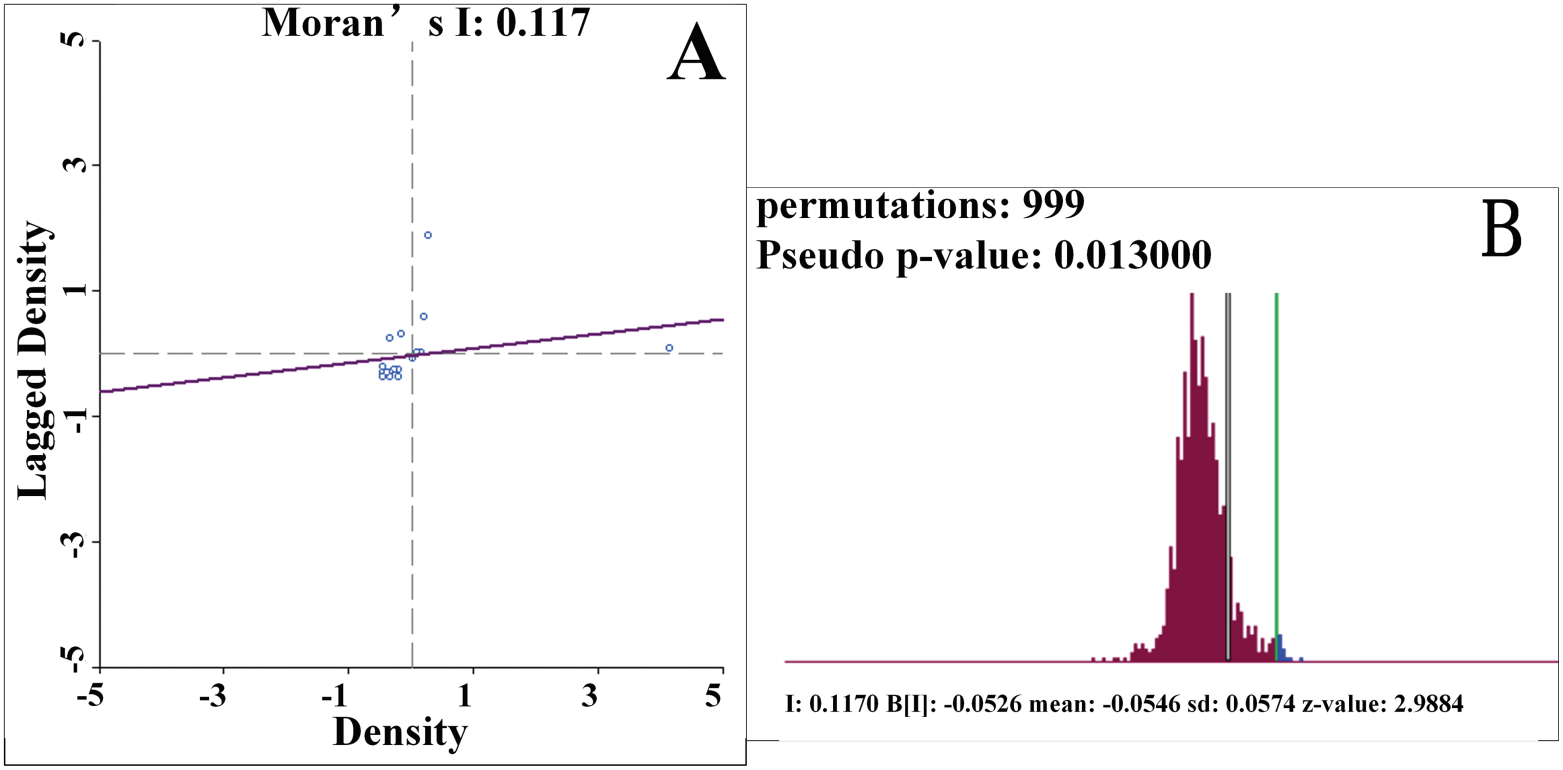
The global Moran’s *I* index (A) and permutation test (B) on the spatial distribution of *Cx. pipiens pallens* density on the level of the subdistrict.

As Fig. 4A shows, the Moran’s *I* value was 0.1170, indicating that the closer the light traps were, the more similar the vector density was. In other words, there was positive spatial autocorrelation among the vector densities. After 999 permutations again in GeoDa, as shown in Fig. 4B, the *P*-value was 0.013, which suggested that there was at least a 95% likelihood that the distribution of Moran’s *I* was not a random distribution but was an empirical distribution. That is, there was statistically significant spatial autocorrelation or spatial clustering among the vector densities of districts. Therefore, a spatial regression model may be suited for the analysis of the relationships between vector density and geographic environmental factors.

### Local Spatial Autocorrelation (LISA) Analysis of Mosquito Density at the Subdistrict Level

**Fig. 5.**
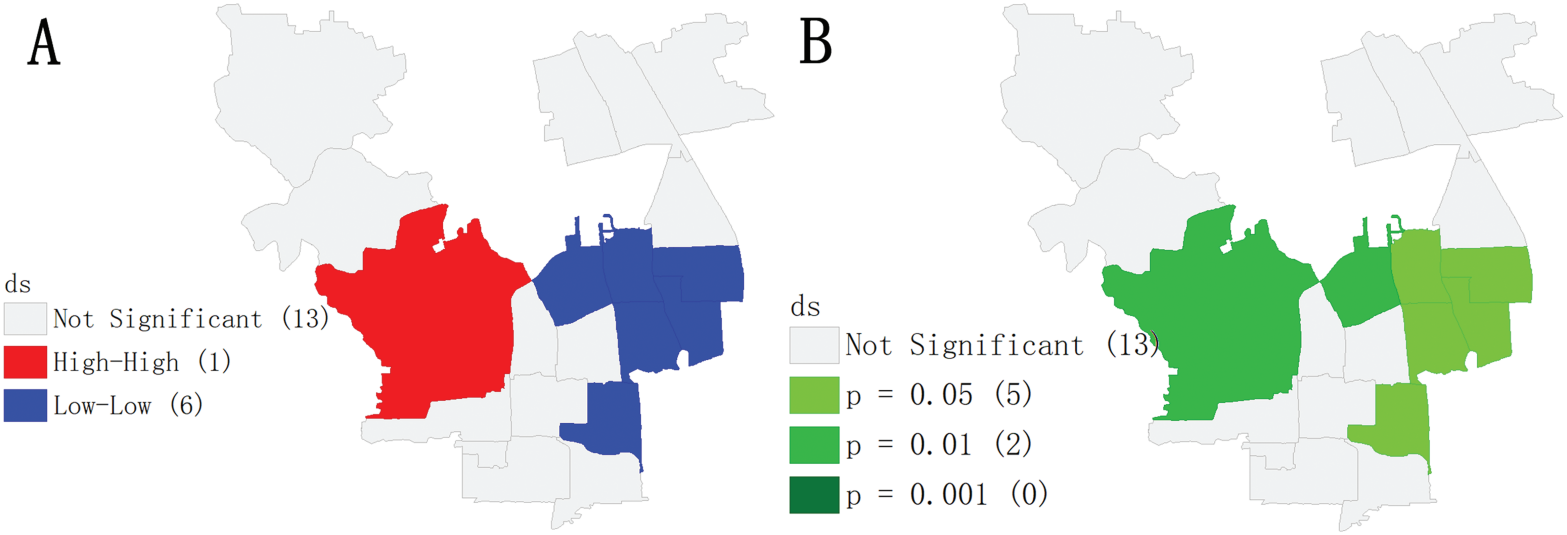
Local cluster maps on the level of subdistrict. A) Cluster map and B) significant level map.

The local cluster map analysis results (Fig. 5) indicated that there were 2 local cluster areas in our study area. One was the high-high cluster center named the Shijiqing subdistrict, lying in the middle of the Haidian district and shown in red in the cluster map (Fig. 5A). Around the Shijiqing subdistrict, the vector density showed a high-high pattern with a significance level of 0.01 (Fig. 5B). That is, the vector density in this area and its surrounding subdistricts is higher than that in other subdistricts in our study area. The other cluster area was the low-low cluster center with 5 subdistricts lying in the middle of our study area and located close to the Beijing city center, which is shown in blue in the cluster map (Fig. 5A). In the significance map, one district was significant at the 0.01 level (Fig. 5B), and the other 5 districts were significant at the 0.05 level (Fig. 5B). Therefore, the vector density in subdistricts around these 6 low-low areas was lower than that in other subdistricts in our study area.

### Spatial Regression of *Cx. pipiens pallens* Density at the District Level

Four factors (MNDWI, NDVI, NDBI, NLI) were included in the regression model using the all-enter way. From large to small, the Abs (Beta) value ranked as MNDWI (4.339), NDVI (2.007), NDBI (2.033) and NLI (0.614). Thus, these 4 factors were introduced into the regression model following the rank as MNDWI-NDVI-NDBI-NLI, which would produce 4 regression model frameworks (Table 1).

**Table 1.** Regression comparison among framework with spatial or nonspatial models

Model	Framework	Regression method	R-squared	AIC
1	MNDWI	Nonspatial	0.645856	151.196
		Spatial-LAG	0.645895	153.194
		Spatial-ERROR	0.652692	150.978
2	MNDWI + NDVI	Nonspatial	0.718835	148.58
		Spatial-LAG	0.741030	149.29
		Spatial-ERROR	0.825059	143.578
3	MNDWI + NDVI + NDBI	Nonspatial	0.718900	150.576
		Spatial-LAG	0.764321	150.164
		Spatial-ERROR	0.845265	143.933
4	MNDWI + NDVI + NDBI + NLI	Nonspatial	0.786173	147.105
		Spatial-LAG	0.833094	145.561
		Spatial-ERROR	0.905976	136.552

For each model framework, 3 regression methods (nonspatial, spatial lag, and spatial error) were applied to obtain the AIC and R-squared values, which resulted in twelve regression models. Among these models, the AIC value ranged from 136.552 (spatial error model of MNDWI + NDVI + NDBI + NLI) to 153.194 (spatial lag model of MNDWI), while the R-squared value ranged from 0.645895 (nonspatial model of MNDWI) to 0.905976 (spatial error model of MNDWI + NDVI + NDBI + NLI). The spatial error model of MNDWI + NDVI + NDBI + NLI produced the lowest AIC value (136.552) and the highest R-squared value (0.905976), indicating that this spatial error model, including all 4 factors, is the best model for the regression of vector density.

### Factor Coefficients of *Cx. pipiens pallens* Density at the District Level

In the coefficient table of the spatial error model with the factors MNDWI + NDVI + NDBI + NLI, MNDWI, and NDVI displayed negative coefficients with the vector density, and NDBI and NLI had positive coefficients with vector density. In the “Probability” row in Table 2, the probabilities of NDVI (Coefficient = −8.79019, *P* = 0.0002) and NLI (Coefficient = 9.90899, *P* = 0.0005) were smaller than 0.05, indicating that these 2 factors significantly affected vector density.

**Table 2.** Coefficient of factors with the density of *Cx. pipiens pallens* on the level of district

Variable	Coefficient	St. error	*z*-Value	Probability
CONSTANT	9.22204	0.570615	16.1616	0.00000
MNDWI	−3.97308	18.1185	−0.219283	0.82643
NDVI	−8.79019	2.38288	−3.68889	0.00023
NDBI	19.8936	14.7292	1.35062	0.17682
NLI	9.90899	2.85492	3.47085	0.00052
LAMBDA	−0.943532	0.110526	−8.53673	0.00000

### Spatial Distribution at the LA Level

**Fig. 6.**
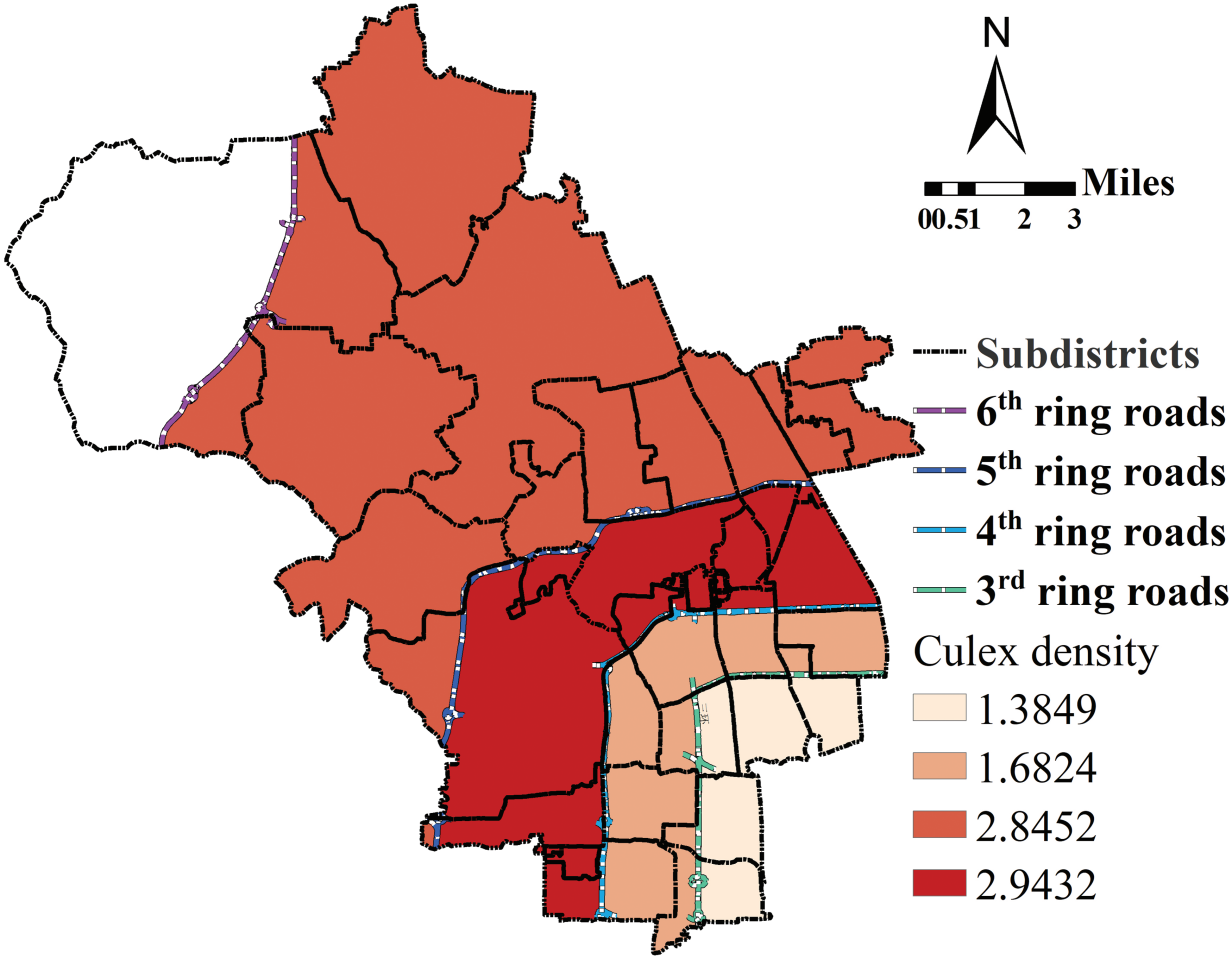
Mosquito density spatial distribution on the level of LA.

As shown in Fig. 6, the density distribution of *Cx. pipiens pallens* at the LA level showed the same “middle peak” pattern as at the subdistrict level. The density of *Cx. pipiens pallens* in 2 LAs (LA1 and LA2) near the city center was the lowest (LA1 = 1.3849 NMPT and LA2 = 1.6824 NMPT). The density of *Cx. pipiens pallens* in the LA laying farthest from the center city was intermediate (LA4 = 2.8452 NMPT), and the *Cx. pipiens pallens* density in the middle LA was the highest density (LA3 = 2.9432 NMPT). Based on the results of one-way ANOVA followed by multiple comparisons of the LSD test, some conclusions could be drawn: first, there were significant mean differences in the density of *Cx. pipiens pallens* among the 4 LAs (*F* = 6.493, *P* = 0.0010); second, there were no significant mean differences in the density of *Cx. pipiens pallens* between LA1 and LA2 (*P* = 0.4877) or between LA3 and LA4 (*P* = 0.8280); third, there were significant mean differences in the density of *Cx. pipiens pallens* between LA1 and LA3 (*P* = 0.0022), LA1 and LA4 (*P* = 0.0025), LA2 and LA3 (*P* = 0.0053), and LA2 and LA4 (*P* = 0.0060).

## Discussion

The environment is considered the essential driver of *Cx. pipiens pallens* distribution in the context of urban areas (Cardo et al. 2018). Thus, the spatial distribution characteristics should result from the interaction between *Cx. pipiens pallens* and environmental factors. In this study, many environmental factors, including the NDVI, MNDWI, NDBI, and light index, were found to be related to the *Cx. pipiens pallens* density. Among these, the relationship of the light index with the density of *Cx. pipiens pallens* had not been previously reported, and thus, this study sheds light on this relationship for the first time. However, NDVI, MNDWI, NDBI and light index have been reported to be closely related to *Cx. pipiens pallens* populations in urban areas (LaBeaud et al. 2008, Roiz et al. 2015). *Culex* species, particularly *Cx. pipiens pallens*, was confirmed to have high adaptation to the urban environment by many studies (Nchoutpouen et al. 2019, Xavier et al. 2019). It is possible that the urban environment possesses special features that contribute to the adaptation of *Cx. pipiens pallens* to urban areas. The environment determines the distribution of mosquito-borne diseases in that it influences the vector–host‒pathogen transmission cycle, including vector distribution, abundance, and diversity (Roiz et al. 2015). Thus, this study sheds light on the control exerted by environmental features on *Cx. pipiens pallens* mosquitoes in the Beijing urban area; that is, the potential risk of mosquito biting and WNV or LF transmission in Beijing could be managed.

The vegetation cover in urban areas, such as grass, shrubs, and forests, could be used by adult mosquitoes as resting and hiding sites or may provide shade for larval breeding waterbodies. Thus, vegetation is a major environmental feature impacting the distribution of *Cx. pipiens pallens* vector mosquito density as well as that of mosquito-borne disease cases. At the subdistrict scale, the NDVI was negatively correlated with the density of *Cx. pipiens pallens* (Table 2, coefficient value = −8.79, *P* = 0.0002). According to previous studies, vegetation or its index (NDVI) should be positively linked to mosquito density. However, the present study found adverse impacts of vegetation on the density of the *Cx. pipiens pallens* mosquito and offers different insights into its ecological distribution pattern in urban settings. Few studies have indicated a negative association between NDVI and WNV incidence (Myer and Johnston 2019) or between LF cases and green space metrics (Huang et al. 2018) in urban settings. From the viewpoint of mosquito-borne disease epidemiology, we could deduce that such a negative association must result from a negative correlation of mosquito vector density with vegetation, although these studies did not provide a direct association between mosquito vector density and vegetation. According to previous studies, *Cx. pipiens pallens* was positively correlated with NDVI or vegetation in natural environments or rural areas. Why was there a negative correlation in urban areas? This correlation discrepancy may originate from the background environment. In the countryside, the natural vegetation is not disturbed by humans, while the vegetation in urban areas receives frequent management from landscape departments. Such management may include planting, pruning, fertilizing, insecticide spraying, and so on. These management practices impose negative effects on the mosquito (including *Cx. pipiens pallens*) population, which must be the first reason for the negative correlation between *Cx. pipiens pallens* and vegetation. In turn, the acreage of vegetation also indicates the urbanization level of the local area. A previous study also revealed that *Cx. pipiens pallens* prefers to breed in polluted waterbodies in residential areas, and the greater the human density, the higher *Cx. pipiens pallens* density. Therefore, the more vegetation acreage there is, the less human resident area there is, which leads to fewer *Cx. pipiens pallens* breeding sites as well as lower *Cx. pipiens pallens* density. Thus, this study provides new insight into the effect of vegetation on *Cx. pipiens pallens* in urban areas and the mosquito’s spatial distribution patterns in urban settings, which can aid the field control of *Cx. pipiens pallens* in a city setting.

In this study, *Cx. pipiens pallens* was negatively correlated with the MNDWI at the subdistrict level, which seems contrary to the popular idea of the link between mosquitoes to waterbodies. The presence of water bodies is a prerequisite breeding condition for all mosquitoes because the mosquito spends 3 development stages in water–eggs, larvae, and pupae. Thus, waterbodies are generally considered a positive factor affecting mosquito density. For example, the precipitation in city areas was found to be positively correlated with the density of the *Cx. pipiens pallens* population (Ha et al. 2021), and positive correlations were also identified between minimum infection rates, vector abundance, and cumulative precipitation (Giordano et al. 2017) because precipitation could produce many waterbodies for the breeding of *Cx. pipiens pallens*. However, the abundance of *Cx. pipiens* was negatively correlated with local modeled surface wetness (Shaman et al. 2002), which showed the same relationship between the waterbody and mosquito density as the present study. Such a negative correlation between waterbodies and *Cx. pipiens pallens* density may originate from the special water environmental features in our study area. Waterbodies that are closely related to various mosquitoes include stagnant water, slow-flow water, ground area water, wastewater, and container water. With regard to *Cx. pipiens pallens*, it prefers polluted waterbodies, and its larval density correlates positively with the ammonia nitrogen in the water (Wang et al. 2020). Therefore, polluted urban rivers provide suitable breeding habitats for *Cx. pipiens pallens* (Ma et al. 2016), and drainages were the most productive habitat type for *Culex* larvae (Liu et al. 2015). For urban rivers, flow velocity and water quality are key features driving the density of *Cx. pipiens pallens* in rivers (Mogi and Sota 1996). The MNDWI only includes surface water bodies such as urban rivers, lakes, and channels, excluding waterbodies in drainages. Thus, there is a negative correlation between the MNDWI and *Cx. pipiens pallens* density in this study revealed a negative link between *Cx. pipiens pallens* density and surface waterbodies. In Beijing, the urban surface water was under strict management by the water department (Yaling et al. 2018); for example, plants and waste in water were cleared, the flow of rivers was promoted, and the level of ammonia nitrogen in water was regulated. Therefore, there is a negative correlation between the MNDWI and *Cx. pipiens pallens* density in this study was reasonable from the viewpoint of environmental entomology. This study not only reveals the negative correlation between surface water and *Cx. pipiens pallens* density but also points out that nonsurface water (drainages, for example) is responsible for the high density of *Cx. pipiens pallens* in the Beijing urban area. In the future, control efforts should focus on the *Cx. pipiens pallens* in drainages or other nonsurface waters based on continued surface water management efforts.

Buildings in urban areas are the major landscape features that affect the population of *Cx. pipiens pallens* in urban areas. In this study, the NDBI, representing the urban built-up landscape, was positively correlated with the abundance of *Cx. pipiens pallens* at the community level. In fact, few studies have revealed a negative effect of the building landscape on *Cx. pipiens pallens* density. In central Iowa, for example, increasing levels of urbanization over time resulted in a dramatic decline in *Cx. pipiens*, and increasing landscape development may also have negative impacts on *Culex* vector populations (Field et al. 2019). In addition, a large number of previous studies have suggested a positive influence of buildings or the built-up landscape on *Cx. pipiens pallens* in urban areas. In North America, *Cx. pipiens pallens* population abundance is positively correlated with human population density, housing unit density, urban land use, and land cover classes, and it is negatively correlated with the age of dwellings (Trawinski and Mackay 2010). In the eastern United States, urban infrastructure was also positively correlated with the abundance of *Cx. pipiens* (Deichmeister and Telang 2011). Therefore, the present study clarifies a similar relationship between the urban built-up landscape and *Cx. pipiens pallens* density. Moreover, our study uncovered the effect of the spatial distance of the built-up landscape on the density, and the built-up landscape located at 800 m around the trapping site correlated positively with the *Cx. pipiens pallens* density. The built-up landscape’s positive link to *Cx. pipiens pallens* density must also depend on its effect on *Cx. pipiens pallens* breeding. In the context of the urban environment, buildings are always accompanied by an underground drainage system construction and sewage flow or retention systems, which would result in increased *Cx. pipiens pallens* density in regions with a higher building index. In this study, we uncovered a correlation between the built-up index with *Cx. pipiens pallens* density, which highlights the need for the control of *Cx. pipiens pallens* in the Beijing urban area. That is, we must pay more attention to *Cx. pipiens pallens* infestations in Beijing land planning since urban development would result in the construction of many buildings.

Light is a key environmental feature affecting the mosquito population, regardless of whether it is natural or artificial in origin (Cardo et al. 2020). In this study, the urban NLI was positively correlated with the abundance of *Cx. pipiens pallens* in the Beijing urban area at the subdistrict scale. In this study, the NLI index is the urban artificial light value, and few studies have uncovered a relationship between urban nighttime light and the density of *Cx. pipiens pallens*. The NLI index was found to be negatively associated with the abundance of *Cx. Pipiens pallens* in light traps (Boze et al. 2021). However, sunlight, as a natural light source, has a negative effect on the breeding of *Cx. Pipiens pallens*, as reported in many studies. For instance, sunny ponds with steep sides and little vegetation generally produce the fewest mosquitoes (Gingrich et al. 2006) and *Cx. pipiens pallens* showed a higher container index in shaded containers than in sunlit containers (Vezzani and Albicocco 2009). In this study, the positive correlation between the urban light index seemed reasonable from the viewpoint of the urbanization level and the *Cx. pipiens pallens* density. Indeed, the urban nighttime light index is a popular index used to represent the urban development level. Therefore, a higher nighttime light value means a higher urban landscape or urban building utilization level. As shown above, *Cx. pipiens pallens* prefers breeding in underground drainages. Thus, the higher the urban landscape building utilization is, the more complex or larger the underground drainage system, which results in a higher *Cx. pipiens pallens* breeding density in local areas. To date, few studies have revealed the relationship between the urban nighttime light index and *Cx. pipiens pallens* density. Moreover, environmental variables estimated by remote sensing and the spatial distribution (presence, abundance, and diversity) of mosquitoes have been applied to WNV and its vector, and remote sensing data provide reliable information for predicting and monitoring mosquito population distribution and abundance (Roiz et al. 2015). Therefore, the present study could aid in estimating the risk of *Cx. pipiens pallens* transmission of WNV in Beijing from the viewpoint of mosquito-borne disease epidemiology.

There are diverse mosquito communities in different ecoregions of cities, and information on how vectors are distributed across urban spaces is vital to successful vector-borne disease and vector management. In the Haidian district of Beijing, the distribution of *Cx. pipiens pallens* density was nonrandom. That is, global and local spatial clusters were detected in the present study. The local Moran’s *I* test revealed 2 spatial subdistrict clusters: the high-high cluster in the Shijiqing subdistrict in the transition zone and the low-low cluster in 6 subdistricts in the core zone. In the past, the distribution of *Cx. pipiens pallens* in Beijing was thought to be spatially random, and there was no consideration of the spatial clustering of *Cx. pipiens pallens*. Therefore, the analysis of the spatial distribution has been a major focus of spread pattern studies among different environment types (Meide et al. 2019, Ting et al. 2021). For the first time, the present study examines the distribution of *Cx. pipiens pallens* from the viewpoint of subdistricts. The distribution of *Cx. pipiens pallens* was diverse in multiple ecoregions of cities (Bradt et al. 2019), and the number and occurrence peak of the mosquitoes were significantly different from those in the other types of residential areas (Chuan-Ming et al. 2017). The present study not only revealed the spatial diversity distribution in the Haidian district but also detected one high-high and one low-low cluster of *Cx. pipiens pallens* density at the subdistrict level. The distribution of the biotic and abiotic environment contributes to spatial and temporal variation in the production of mosquito vectors in cities (Gardner et al. 2013). Thus, the spatial clustering of *Cx. pipiens pallens* density in the Haidian district may result from the spatial environment in the subdistricts from the viewpoint of vector ecology. In turn, there was a heterogeneous environmental pattern in these subdistricts, and nearby subdistricts shared similar environmental characteristics. These 2 reasons lead to the spatial spreading pattern of the vector among the subdistricts in the present study area. This study highlights the spatial distribution pattern of *Cx. pipiens pallens* in a Beijing district, as well as the high-high and low-low clusters among subdistricts, which could aid in the vector management of *Cx. pipiens pallens* in subdistricts and in vector resource allocation for the Haidian district.

In this study, the communities and subdistricts with the highest density of *Cx. pipiens pallens* were in the middle LA of Haidian district, while there was a lower *Cx. pipiens pallens* density in the communities and subdistricts in central LA. Moreover, the *Cx. pipiens pallens* density at the community and subdistrict levels could be modeled by linear and autoregression spatial models. Urban areas benefit urban-adapted mosquito species globally, and the urbanization level of diverse areas is a major impact factor on *Culex* phenology density in city environments (Townroe and Callaghan 2014). Therefore, the rapid growth of urbanization would result in an increased risk of *Cx. pipiens pallens* infestation and vector-borne disease transmission (Yuan et al. 2012, Mwakitalu et al. 2013).

In this study, the urban-level indices, such as the NDBI and NLI, were also found to be positively correlated with *Cx. pipiens pallens* density. On this point, the present study agrees with a previous study. At the urbanization level scale, there is a decrease in urbanization level from the center to the outside periphery (the center, transition zone, and suburban area) in the Haidian district administration region. Accordingly, the density of *Cx. pipiens pallens* should decrease with the decrease in the urbanization level. However, the present study reveals that *Cx. pipiens pallens* was primarily distributed in the transition zone of the Haidian district, with a lower-density cluster in the center districts. Thus, the higher density in the transition area may also originate from its particular environmental features or urban management level. The rapid growth of cities combined with limited economic resources often results in informal settlements and slums with favorable conditions for the proliferation of vectors of LF (Mwakitalu et al. 2013). However, if the urban area is well managed, the environment could suppress the density of *Cx. pipiens pallens* and its transmitted disease, and high densities of *Cx. pipiens pallens* would generally be found in other areas of the city with lower management levels (Erlanger et al. 2005). In the present study area, the closer to the center of Beijing, the more administrators would have the ability to conduct environmental management, which would negatively breed and decrease the density of *Cx. pipiens pallens*. Additionally, the negative correlation of the NDVI and MNDWI to the *Cx. pipiens pallens* density revealed in the present study confirmed our discussion on this point. However, urbanization could facilitate the breeding of *Cx. pipiens pallens* in urban areas; this study revealed that urban environmental management could decrease the *Cx. pipiens pallens* density in the urban center region. Furthermore, the spatial distribution pattern of *Cx. pipiens pallens* at the community, subdistrict, and LA scales found could aid in the urban control of *Cx. pipiens pallens* in Beijing. Finally, *Cx. pipiens pallens* density could be modeled with a particular correlation of environmental features on diverse spatial scales, such as the MNDWI and NDBI at the community level and NLI and NDVI at the subdistrict level, which would also contribute to scientific and precise control of *Cx. pipiens pallens* in the urban area of Beijing.

This study examined the spatial distribution pattern and environmental correlations of *Cx. pipiens pallens* density in the urban setting of the Haidian district of Beijing. For the first time in this study, the spatial distribution characteristics of *Cx. pipiens pallens* at the community, subdistrict, and LA levels as well as the environmental correlations at the community and subdistrict scales, are described. The Haidian district, our study area, has a topography similar to Beijing’s, covers the CFCA and UFDA, has a GDP ranking first among districts in Beijing, and supports the second-largest population in Beijing. Thus, this study makes sense not only for the Haidian district but also for Beijing City from the viewpoint of public health, the environment and the economy. First, this study suggests that the *Cx. pipiens pallens* density could be managed via human interference in the urban environment (for example, on vegetation, water bodies, and underground drainages), although many studies have demonstrated the positive relationship of the urbanization level with *Cx. pipiens pallens* density. Second, with the advantage of 3S technology, we revealed the correlated environmental features and fit the spatial autoregression and linear regression models at the subdistrict and community levels, which could aid in the prevention and control of *Cx. pipiens pallens* in the larger urban area of Beijing. Nevertheless, there are 2 limitations that should be addressed in future studies. First, there were few LA samples in this study, so we could not perform the correlation and model analysis at the LA level. Second, the light trapping sites were located in the residential area in the Haidian district, which produced a certain degree of environmental blankness to explain the *Cx. pipiens pallens* spatial distribution, and more environmental types should be included in the future.
